# Exploring life engagement from the perspective of patients with major depressive disorder: a study using patient interviews

**DOI:** 10.1186/s41687-022-00517-z

**Published:** 2022-10-12

**Authors:** François Therrien, Stine R. Meehan, Catherine Weiss, Jennifer Dine, T. Michelle Brown, Erin M. MacKenzie

**Affiliations:** 1 CNS Medical Affairs, Otsuka Canada Pharmaceutical Inc., 2250 Bd Alfred Nobel, Saint-Laurent, QC H4S 2C9 Canada; 2 H. Lundbeck A/S, Valby, Denmark; 3 Otsuka Pharmaceutical Development & Commercialization Inc., Princeton, NJ USA; 4 RTI Health Solutions, Research Triangle Park, NC USA; 5 Lundbeck Canada Inc., Saint-Laurent, QC Canada

**Keywords:** Major depressive disorder, Patient reported outcome measures, Self report, Interview, Patient participation, Patient life engagement

## Abstract

**Background:**

Patient-reported outcomes can measure health aspects that are meaningful to patients, such as ‘life engagement’ in major depressive disorder (MDD). Expert psychiatrists recently identified ten items from the Inventory of Depressive Symptomatology Self-Report (IDS-SR) that can be used to measure patient life engagement. This study aimed to explore the concept of patient life engagement and provide support for the IDS-SR_10_ Life Engagement subscale from the patient perspective.

**Methods:**

Semi-structured video interviews were conducted with adults with MDD in the United States. Patients were asked if they ever felt engaged with life, and how this affected their feelings, activities, socializing, and thoughts. Then, patients discussed the ten expert-selected IDS-SR items, and rated the relevance of all 30 items to patient life engagement on a 4-point scale.

**Results:**

Patients (N = 20) understood the ‘engaged with life’ concept and could provide examples from their own lives, such as increased energy/motivation (100%), being more social/spending time with others (85%), being more communicative (80%), and having better mood (75%). Nineteen patients (95%) indicated that all ten IDS-SR_10_ Life Engagement items were relevant to patient life engagement, and nine of the ten items had a mean score ≥ 3 (moderately relevant). Four additional items (all relating to mood) also scored ≥ 3.

**Conclusions:**

Patients found the concept of life engagement to be important and relatable, and confirmed the IDS-SR_10_ captures the defining non-mood-related aspects of patient life engagement. This research supports the relevance of patient life engagement as a potential clinical outcome beyond core mood symptoms, and the use of the IDS-SR_10_ Life Engagement subscale in patient-oriented research.

**Supplementary Information:**

The online version contains supplementary material available at 10.1186/s41687-022-00517-z.

## Background

The treatment goals of patients with major depressive disorder (MDD) often differ from those of the clinician, with patients prioritizing positive mental health outcomes such as optimism, vigor and self-confidence, meaningfulness of life, life enjoyment, and satisfaction with oneself, over improvement of depressive symptomatology and functioning [1, 2]. To ensure that clinical research is relevant to patients and that appropriate outcome measures are selected, regulatory bodies such as the United States Food and Drug Administration and the European Medicines Agency encourage the incorporation of patient experience data into clinical trial design [3–6]. In addition, the use of patient-reported outcomes (PROs) is recommended to evaluate the effects of a drug from the patient’s perspective, alongside the clinician’s assessment [4–9].

Recently, the study sponsors received unsolicited patient experience data from patient call centers, together with feedback from health care professionals, which described benefits of a depression treatment (adjunctive brexpiprazole) that were not fully captured by existing terminology or clinical trial outcomes [10]. The benefits encompassed positive health aspects relating to cognition (including ‘hot’ cognition, i.e., cognition colored by emotion), vitality, motivation and reward, and the ability to feel pleasure, reflecting the functional outcomes of life fulfillment, well-being, and participation in valued and meaningful activities [10, 11]. To capture these benefits, a panel of expert psychiatrists devised a framework for patient life engagement that comprised four domains: emotional (affect/mood), physical (energy/motivation), social (involvement/interest) and cognitive (alertness/thinking) [10]. Patient life engagement is, therefore, a broad construct that aligns with patient-reported indicators of treatment effectiveness in depression [10, 12], and which differs from previously defined concepts in mental health. For example, psychological well-being (comprising self-acceptance, purpose in life, autonomy, positive relations with others, environmental mastery, and personal growth [13, 14]) and social well-being (comprising social coherence, actualization, integration, acceptance, and contribution [15]) do not fully capture the energy and alertness aspects of patient life engagement. Similarly, the ‘CHIME’ (Connectedness, Hope and optimism about the future, Identity, Meaning in life, and Empowerment) conceptual framework for personal recovery in mental illness [16] does not fully cover the energy and cognitive aspects of patient life engagement. Patient life engagement overlaps with the concept of wellness; however, wellness places less emphasis on social and cognitive aspects of health, which are considered lower priorities than the ability to act independently (i.e., being in control of one’s emotions and decisions), getting through the day, having influence over events, and having purpose in life [17]. Patient life engagement also differs from life satisfaction, with the latter being an overall assessment of feelings and attitudes to life, i.e., a “cognitive, judgmental process” predominantly in the emotional domain [18, 19]. Finally, patient life engagement has a bidirectional relationship with patient functioning: as an individual becomes more engaged with life (e.g., increased energy, alertness, interest), their functionality increases (e.g., greater role performance, ability to perform daily activities), and vice versa [20].

A recent systematic literature review identified 49 validated PROs, such as the Warwick–Edinburgh Mental Well-Being Scale [21], that can capture aspects of the patient life engagement framework in the field of mental health [22]. However, the majority of identified PROs captured only a single aspect of patient life engagement—most commonly motivation and reward, pleasure, and psychological well-being [22]. Other PROs, such as the 93-item Quality of Life Enjoyment and Satisfaction Questionnaire (Q-LES-Q), are sufficiently comprehensive that most aspects of patient life engagement are covered [23]. However, many items in the Q-LES-Q are not directly relevant to patient life engagement (e.g., physical health, economic status), as this measure was designed to capture enjoyment and satisfaction in multiple specific aspects of daily functioning, rather than life engagement [23]. Thus, there is a need for a PRO that can measure patient life engagement in psychiatric clinical trials in a manner that is comprehensive and yet focused. Recently, experts in psychiatry adapted an existing PRO—the 30-item Inventory of Depressive Symptomatology Self-Report (IDS-SR) [24]—for this purpose, by selecting ten items from the scale that represent patient life engagement beyond the core symptoms of depression (i.e., core mood items were deliberately excluded) [25]. The ten selected items were: response of your mood to good or desired events, concentration/decision making, view of myself, view of my future, general interest, energy level, capacity for pleasure or enjoyment (excluding sex), interest in sex, feeling slowed down, and interpersonal sensitivity. These ten items (plus an additional three items) clustered in a principal component analysis that incorporated data from over a thousand patients with MDD [25], supporting the grouping of the items into an exploratory subscale termed the ‘IDS-SR_10_ Life Engagement subscale’.

The aims of the present study were to explore the concept of life engagement and its relevance in MDD from the perspective of patients for the first time; and to obtain patient confirmation of the ten expert-selected items in the IDS-SR_10_ Life Engagement subscale (i.e., content validity), to support its use in research and clinical practice.

## Methods

### Study design and patients

A protocol was developed by RTI Health Solutions in collaboration with the study sponsors. Study materials were reviewed and approved by RTI International’s institutional review board before any potential participants were informed or recruited for the study, and patients provided verbal informed consent to participate.

The study comprised semi-structured, 60-min video interviews with adults with MDD in the United States. For each interview, a cognitive interviewing approach was used [26]. Specifically, participants were asked to provide feedback on the concept of patient life engagement by spontaneously describing attributes relevant to their interpretation of patient life engagement, as well as by responding to specific probes designed to elicit feedback on characteristics of the patient life engagement framework (i.e., emotional, physical, social, and cognitive domains) [10]. Participants were also asked to provide feedback on the relevance and interpretability of IDS-SR items, in order to determine their content validity and representativeness of patient life engagement, in alignment with the overarching cognitive interviewing design [26].

Patients were eligible if, according to their answers in a screening interview, they were aged ≥ 18 years, had a clinician-provided diagnosis of MDD, had their first depressive symptoms ≥ 1 year ago, had a depressive episode within the past 3 months, were currently taking a prescription antidepressant, and did not have another specified diagnosis (Alzheimer’s disease, dementia, schizophrenia/schizoaffective disorder, or bipolar disorder). Patients needed to have access to a computer/tablet with a camera, to have broadband or high-speed internet, and to be able to read and write in English. While there were no recruitment quotas, recruiters aimed to enroll a mixture of sexes, races, educational levels, and ages. In addition to assessing eligibility, the screening interview collected patient demographics.

### Video interviews

Each patient interview was conducted by two experienced qualitative researchers at RTI Health Solutions (Dr. Brown and Dr. Dine) via an online video platform (Webex). Based on a semi-structured interview guide, one interviewer took the lead, while the second interviewer made field notes and ensured that no topic was missed. Interviews were conducted between June 8 and June 17, 2020, and were audio recorded and transcribed.

The aim of the interview was to gather information about patients’ perceptions and experiences with depression, including improvements associated with successful treatment, as well as to review and provide feedback on the relevance of IDS-SR items. After a brief introduction to the study, patients were asked to comment on the impact of the ongoing COVID-19 pandemic on their daily life and mood, in order to (a) acknowledge the pandemic, and (b) evaluate the potential impact of the pandemic on the results of this study. In addition, patients were asked about their depressive history (age at MDD diagnosis, and when their most recent episode occurred), and to discuss their current mood (“How do you feel today?”).

**Fig. 1:**
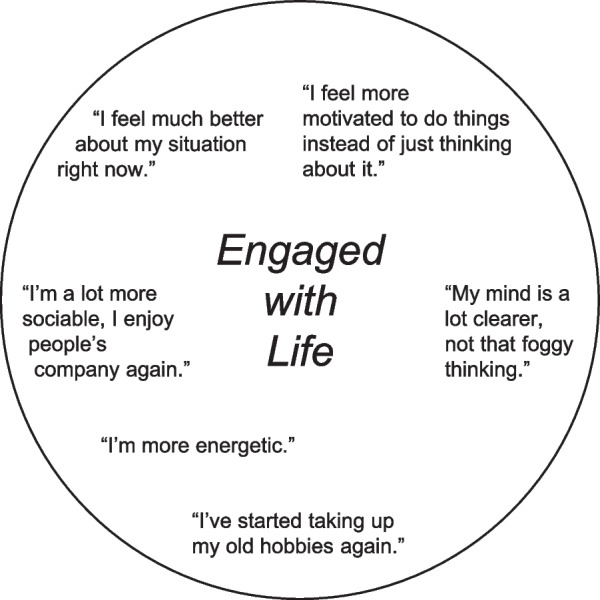
“Engaged with life” handout

Next, patients were introduced to the concept of patient life engagement, via a handout with illustrative quotations derived from clinical trial exit interviews (Fig. 1) [10]. Having considered the handout, patients were asked if they had ever felt engaged with life, and to describe how this felt. If not spontaneously reported, patients were asked specific questions in four domains: feelings, including motivation, energy, and interest (emotional); daily activities (physical); involvement or communication with others (social); and thinking (cognitive).

Finally, patients considered a worksheet comprising a list of the 30 IDS-SR items, which had been modified to (a) reorder the items so that the ten proposed patient life engagement items came first (since an objective of the study was to confirm the experts’ selection of these items) and (b) provide examples for certain items so that patients could better understand the terms (e.g., for interpersonal sensitivity: “feeling rejected, criticized, or hurt by others”). The modified IDS-SR worksheet is provided in Additional file 1: Appendix 1. Patients were asked to discuss the relevance of the first ten items to being engaged with life, and whether they could think of any aspects of patient life engagement that were missing from these ten items. Then, patients were asked to rate all 30 items according to their relevance to being engaged with life, using a 4-point scale: 1 (not at all relevant), 2 (a little relevant), 3 (moderately relevant), or 4 (very relevant). After reviewing all items, patients were asked again if any aspects of patient life engagement were missing.

A copy of the interview guide is provided in Additional file 1: Appendix 2.

### Statistical analysis

In alignment with the cognitive interviewing approach, participant feedback was iteratively reviewed to assess the relevance of each aspect of patient life engagement (and interpretability of each IDS-SR item), as well as to identify any new or missing aspects to support the refinement of the patient life engagement framework and inform the refinement/selection of the IDS-SR items that would be used to assess patient life engagement. Specifically, interview transcripts and field notes were used to qualitatively identify, characterize, and summarize themes of the patient life engagement discussion (a thematic analysis). An initial thematic coding framework was developed based on the interview guide topics, which was applied to the data by assigning codes to segments of text in the interview transcripts, and modified iteratively to accommodate information arising from the analysis (i.e., any new aspects identified with each interview). Then, the number and percentage of patients who reported each theme was determined. Patient ratings of the relevance of IDS-SR items were summarized using descriptive statistics. No statistical comparisons were conducted due to the qualitative nature of the study and small sample size.

## Results

### Patients

Twenty patients were interviewed, with a mean age of 43 years (range: 20–70 years) and a mean time since MDD diagnosis of 14.8 years (range: 2–50 years). Approximately half of the patient sample had experienced symptoms of depression within the past 2 weeks (Table 1).

**Table 1:** Patient characteristics at screening

Characteristic^a^	N = 20
Demographic characteristics	
Age (years), mean (SD)	43 (15.2)
Female	10 (50.0)
Race	
White	10 (50.0)
Black or African American	7 (35.0)
Hispanic	2 (10.0)
Asian	1 (5.0)
Education level	
Less than college degree	8 (40.0)
College degree or higher	12 (60.0)
Clinical characteristics	
Time since MDD diagnosis (years), mean (SD)	14.8 (12.7)
Most recent depressive symptoms	
Within the past 2 weeks	11 (55.0)
More than 2 weeks ago	9 (45.0)

MDD, major depressive disorder; SD, standard deviation

^a^Data are n (%) unless otherwise specified

At the time of the interview, most participants commented that they felt good or that it was a good or typical day; a few participants reported experiencing a low mood at the time of the interview, which was consistent with previous days. Individuals who reported experiencing low mood at the time of the interview were primarily those who reported more severe or frequent depressive symptoms (e.g., those who reported experiencing more bad days than good days in general, or who had not experienced a break in their depressive episode in months to years).

The most frequently reported current medications for depression were venlafaxine and sertraline (each n = 4; 20%). Others were desvenlafaxine (n = 3; 15%); trazodone, citalopram, quetiapine, paroxetine, escitalopram, aripiprazole, and fluoxetine (each n = 2; 10%); and duloxetine (n = 1; 5%). Almost one third of patients (n = 6; 30%) reported taking two concurrent treatments for their depression.

### Impact of the COVID-19 pandemic

For most patients, the COVID-19 pandemic had a negative impact on their daily life and mood. With regard to daily life, patients commonly reported loss of work and financial hardship, being unable to spend time with family or friends, and needing to adjust to new routines such as homeschooling children and wearing personal protective equipment. With regard to mood, several patients attributed their last depressive episode to the onset of the COVID-19 pandemic. Patients commonly reported that their mood was more variable or more negative during the pandemic, and that they were experiencing increased stress or anxiety. Nonetheless, at the time of the interview, most patients reported having a good or typical day, and therefore the pandemic was not thought to have impacted the findings of the present study.

### Exploration of the patient life engagement concept

Overall, patients said that the ‘engaged with life’ concept was clear, it resonated with them, and they were able to provide examples from their own lives reflecting the emotional, physical, social, and cognitive domains. The most frequently reported aspects of patient life engagement (reported by at least half of the sample) are listed in Table 2. When engaged with life, patients reported feeling more energized and motivated to perform tasks of all kinds, taking greater interest in what they and other people around them were doing, having a more positive outlook on life, and being able to think through problems with greater clarity. Furthermore, when engaged with life, patients thought about activities in which they wanted to participate, actively carried out these activities, found pleasure in them, and were motivated to continue to seek out activities they found enjoyable. Increased socialization was also a frequently reported aspect.

**Table 2:** Most frequently reported aspects of patient life engagement

Aspect reported by ≥ 10 patients^a^	N = 20
Increased energy/motivation	20 (100.0)
More social; spending time with others (e.g., family time, going out with friends)	17 (85.0)
More communicative (e.g., more talkative, initiates conversation)	16 (80.0)
Better mood (e.g., feel better, positive, happy, upbeat)	15 (75.0)
Improved focus, attention, or concentration	11 (55.0)
Doing enjoyable activities (e.g., hobbies, going to the theater, travel)	10 (50.0)
More hopeful/positive outlook about the future	10 (50.0)
Improved decision making (e.g., able to decide, faster/better decision making)	10 (50.0)

^a^Data are n (%)

### Relevance of IDS-SR items to patient life engagement

#### Discussing the IDS-SR_10_ Life Engagement subscale

Nineteen of the 20 patients (95%) agreed that all ten IDS-SR_10_ Life Engagement items were relevant to their perspective of patient life engagement. The remaining patient reported that one item, ‘feeling slowed down’, was not relevant to patient life engagement because this patient reportedly always “takes things slow”.

When asked if any important aspects of patient life engagement were missing from the IDS-SR_10_ Life Engagement subscale, 11 patients (55%) reported at least one missing aspect: sleep problems (n = 3; 15%); general health/well-being, and anxiety (each n = 2; 10%); and restlessness, substance abuse, spirituality, problems with appetite, maintaining a positive environment, and hobbies (each n = 1; 5%). When asked why the missing aspects were relevant to patient life engagement, patients frequently reported that these aspects were important to how they felt when they were depressed or when their symptoms of depression were improving, not necessarily specific to when they were engaged with life.

#### Rating all 30 IDS-SR items

All ten expert-identified patient life engagement items received high patient scores for relevance to being engaged with life (Table 3). Nine of the ten patient life engagement items had a mean score ≥ 3, indicating that they were ‘moderately relevant’ to ‘very relevant’. The exception was ‘interpersonal sensitivity’, scoring 2.9.

**Table 3:** Patient ratings of the relevance of IDS-SR items to life engagement

Item (original IDS-SR numbering)^a^	Mean (SD) relevance rating^b^
*10. The quality of your mood*	3.7 (0.5)
**21. Capacity for pleasure or enjoyment (excluding sex)**	3.7 (0.7)
**20. Energy level**	3.7 (0.8)
**8. Response of your mood to good or desired events**	3.6 (0.8)
**15. Concentration/decision making**	3.6 (0.9)
**19. General interest**	3.5 (0.8)
**17. View of my future**	3.4 (0.9)
**23. Feeling slowed down**	3.3 (0.9)
*5. Feeling sad*	3.3 (1.0)
**16. View of myself**	3.3 (1.0)
7. Feeling anxious or tense	3.2 (1.1)
**22. Interest in sex**	3.0 (1.0)
6. Feeling irritable	3.0 (1.2)
**29. Interpersonal sensitivity**	2.9 (0.9)
*30. Leaden paralysis/physical energy*	2.9 (1.2)
2. Sleep during the night	2.9 (1.1)
1. Falling asleep	2.7 (1.0)
24. Feeling restless	2.6 (1.3)
12. Increased appetite	2.5 (1.3)
4. Sleeping too much	2.5 (1.4)
26. Other bodily symptoms	2.5 (1.3)
9. Mood in relation to the time of day	2.4 (1.2)
18. Thoughts of death or suicide	2.3 (1.4)
27. Panic/phobic symptoms	2.3 (1.3)
13. Decreased weight (within the last 2 weeks)	2.2 (1.3)
3. Waking up too early	2.2 (1.0)
11. Decreased appetite	2.0 (1.2)
14. Increased weight (within the last 2 weeks)	2.0 (1.2)
25. Aches and pains	2.0 (1.1)
28. Constipation/diarrhea	1.6 (0.8)

IDS-SR, Inventory of Depressive Symptomatology Self-Report; SD, standard deviation

^a^Bold text denotes the ten life engagement items selected by a panel of expert academic psychiatrists; italicized text denotes the three additional items that clustered with the ten selected items in a principal component analysis [25]

^b^Response options: 1 = not at all relevant; 2 = a little relevant; 3 = moderately relevant; 4 = very relevant

Four additional items (all relating to mood) also had a mean relevance score ≥ 3, including two of the three items that clustered with the expert-selected items in a principal component analysis—‘the quality of your mood’ and ‘feeling sad’ (Table 3) [25]. The third item that clustered in the principal component analysis, ‘leaden paralysis/physical energy’, scored 2.9. The other two mood items scoring ≥ 3 that did not cluster in the principal component analysis were ‘feeling anxious or tense’ and ‘feeling irritable’.

In general, items with mean relevance < 3 were related to sleep, appetite, weight, and other bodily symptoms (Table 3).

When asked if any important aspects of patient life engagement were missing from the 30 IDS-SR items, 8 patients (40%) reported at least one missing aspect: socialization (n = 5; 25%); spirituality (n = 2; 10%); and hobbies, working, good or bad dreams, getting things done, and overall mental health (each n = 1; 5%). The patients who reported socialization as missing acknowledged that other IDS-SR items (e.g., general interests) might address this point, but said that existing items were not specific enough to socialization.

## Discussion

This is the first study to collect patient experience data via specific discussions with patients on the concept of life engagement. Interview data can provide direct insights into patients’ needs and treatment goals, and patient perspectives on clinical trial design and endpoints, including the development of new PROs and subscales. In the present discussions, patients with MDD found life engagement to be important and relatable. As expected, patient descriptions of life engagement were based on their own experiences, meaning that one patient’s definition varied from the next. Nonetheless, all 20 patients reported having increased energy/motivation when engaged with life; the next most common experiences were spending time with family and friends, and being more communicative. Overall, patient definitions and experiences were consistent with the four domains of patient life engagement previously defined by experts in psychiatry (emotional, physical, social, and cognitive) [10], suggesting that the existing patient life engagement framework successfully captures the patient perspective.

With regard to the exploratory IDS-SR_10_ Life Engagement subscale, there was strong concordance between items selected by experts, items that clustered in the principal component analysis, and items rated as relevant to life engagement by patients (Table 4) [25]. The only item from the subscale that patients did not rate as moderately relevant or higher (≥ 3) was ‘interpersonal sensitivity’, which was rated 2.9. This could be an issue of perspective: interpersonal sensitivity as typically defined (the ability to accurately assess others’ abilities, states, and traits from nonverbal cues [27]) is a social skill relevant to patient life engagement, whereas the IDS-SR definition (feeling rejected, criticized, or hurt by others [28]) considers the patient’s own feelings, not their ability to interpret the feelings of others, and thus may be less relevant to patient life engagement.

**Table 4:** Summary of expert, PCA and patient relevance of IDS-SR items to life engagement

Item (original IDS-SR numbering)	Associated with life engagement?		
	Expert^a^	PCA^b^	Patients^c^
8. Response of your mood to good or desired events	✓	✓	✓
15. Concentration/decision making	✓	✓	✓
16. View of myself	✓	✓	✓
17. View of my future	✓	✓	✓
19. General interest	✓	✓	✓
20. Energy level	✓	✓	✓
21. Capacity for pleasure or enjoyment (excluding sex)	✓	✓	✓
22. Interest in sex	✓	✓	✓
23. Feeling slowed down	✓	✓	✓
29. Interpersonal sensitivity	✓	✓	
5. Feeling sad		✓	✓
10. The quality of your mood		✓	✓
30. Leaden paralysis/physical energy		✓	
6. Feeling irritable			✓
7. Feeling anxious or tense			✓

IDS-SR, Inventory of Depressive Symptomatology Self-Report; MDD, major depressive disorder; PCA, principal component analysis

^a^Items selected by a panel of expert academic psychiatrists [25]

^b^Items that clustered together in a PCA [25]

^c^Items with a mean relevance score ≥ 3 (moderately to very relevant) as rated by patients with MDD

In addition, patients found four mood items (including ‘the quality of your mood’, which rated the highest of all items) to be relevant to patient life engagement, two of which had clustered with the expert-selected items in the principal component analysis (Table 4). Of note, the experts aimed to select items that represented patient life engagement beyond the core symptoms of depression [25], and thus mood items were deliberately excluded. In contrast, patients largely defined life engagement based on their own experiences with depression and their mood when feeling engaged with life, and thus may not be able to distinguish life engagement from core symptoms. Taking these considerations into account, mood items may represent concurrent, rather than defining, characteristics of the patient during times of being engaged with life. Nevertheless, since improved mood and life engagement are overlapping features of improvement from depression, it may be relevant to include some mood items in the patient life engagement subscale. Additional work is underway to compare patient outcomes based on the different sets of items selected by experts and patients. Specifically, a Canadian Phase 4, multicenter, open-label, interventional study in patients with MDD (ClinicalTrials.gov identifier: NCT04830215) included endpoints based on three sets of items: (1) change in IDS-SR_10_ Life Engagement subscale score (co-primary endpoint); (2) change in patient-selected IDS-SR life engagement items (the 13 items rated as most relevant to patient life engagement in the present study); and (3) change in the IDS-SR life engagement items selected by both clinicians and patients (9 items).

Several patients suggested aspects of life engagement that they believed to be missing from the IDS-SR or IDS-SR_10_, which fell into two categories: (1) aspects that are not directly relevant to patient life engagement, but are reflections of a patient’s mood or behavior when not experiencing symptoms of depression (e.g., sleep problems and general health/well-being); and (2) aspects that are directly relevant to patient life engagement, but are partly encompassed by other IDS-SR items (e.g., socialization and hobbies can be captured by the items ‘general interest’ and ‘capacity for pleasure or enjoyment’). Thus, the patient-suggested additions are either beyond the scope of the IDS-SR_10_ Life Engagement subscale, or are already covered to some extent. There may, however, be benefit in adding a specific item on socialization/communication to the subscale, since this domain is included in the patient life engagement framework [10], as well as being among the most frequently reported aspects relevant to patient life engagement in the open-ended discussions with patients.

Overall, this research suggests that patient-rated life engagement is worthy of evaluation in research and clinical practice, since it represents a meaningful outcome to patients beyond improvement of the core symptoms of MDD. The patients’ acceptance of the items of the IDS-SR_10_ Life Engagement subscale, together with the expert psychiatrist consensus, indicates that the PRO has face validity (i.e., the subscale is understandable and perceived as relevant by patients with MDD), and is suggestive of content validity (i.e., the subscale includes all relevant aspects of patient life engagement—except, potentially, aspects of mood and socialization/communication—and excludes irrelevant factors) [29]. However, the subscale’s construct validity still needs be quantitatively evaluated by determining convergence with related psychosocial measures, as well as divergence with unrelated measures [29].

As a largely qualitative interview study intended to explore the concept of patient life engagement and support content validity, the sample size of 20 participants was fit for purpose. Specifically, the sample size was deemed adequate as no new aspects relevant to patient life engagement were identified upon completion of the last interview (i.e., data saturation). However, the study is limited in that the sample comprised patients who self-reported that they had a clinician-provided diagnosis of MDD (diagnoses were not verified). Patients were required to be currently taking a prescription antidepressant, which helped to confirm the diagnosis, although this requirement may have excluded patients with milder depression who received psychotherapy but not pharmacotherapy. Although current symptom severity was not collected, approximately half of the sample reported having depressive symptoms in the past 2 weeks. The lack of a baseline severity measure, reflecting the qualitative nature of the study, complicates the generalizability of the results to the general population with MDD. With regard to the rating of IDS-SR items, there was a possible bias towards higher ratings for the ten expert-selected items, as patients were presented with these items first and made aware that the items were already deemed relevant. This bias should be considered in the context of the objective of the study, which was to confirm the existing selection of items, not to generate a new set of items. The study used video interviews, rather than in-person interviews, which has been shown to reduce depth of discussion by a modest amount, and may exclude patients with low technical literacy [30]. Finally, questionnaire answers in general may be associated with recall bias, meaning that participants may not accurately or fully remember their life experiences.

Since this study was conducted during the 2020 COVID-19 pandemic, questions were asked to determine the impact of the pandemic on the patients’ answers. Most patients reported that the pandemic had negatively affected their daily lives and mood. However, on the day of the interview, patients were generally in good mental health, and were able to describe times when they felt engaged with life. Consequently, patient feedback does not suggest that the pandemic influenced the results of this study.

## Conclusions

Based on 20 qualitative interviews with patients with MDD, the patient life engagement construct resonated as important and was easily understood. Patients were able to relate to all aspects of life engagement across emotional, physical, social, and cognitive domains. While patients considered 13 IDS-SR items to be most relevant to patient life engagement, the ten expert-identified items were found to capture the defining non-mood-related aspects of patient life engagement, supporting the subscale’s content validity. This research supports the relevance of patient life engagement as a potential clinical outcome beyond core mood symptoms, and the use of the IDS-SR_10_ Life Engagement subscale in patient-oriented research.

## Supplementary Information

**Additional file 1. Appendix 1**. Worksheet to capture patient ratings of relevance to life engagement for each item in the modified Inventory of Depressive Symptomatology Self-Report (IDS-SR).** Appendix 2**. Interview guide.

## Data Availability

To submit inquiries related to Otsuka clinical research, or to request access to individual participant data (IPD) associated with any Otsuka clinical trial, please visit https://clinical-trials.otsuka.com/. For all approved IPD access requests, Otsuka will share anonymized IPD on a remotely accessible data sharing platform.

## Abbreviations

IDS-SR: Inventory of Depressive Symptomatology Self-Report
MDD: Major depressive disorder
PRO: Patient-reported outcome

## References

[1] Zimmerman M, McGlinchey JB, Posternak MA, Friedman M, Attiullah N, Boerescu D (2006). How should remission from depression be defined? The depressed patient’s perspective. Am J Psychiatry.

[2] Demyttenaere K, Donneau AF, Albert A, Ansseau M, Constant E, van Heeringen K (2015). What is important in being cured from depression? Discordance between physicians and patients (1). J Affect Disord.

[3] Kieffer CM, Miller AR, Chacko B, Robertson AS (2020). FDA reported use of patient experience data in 2018 drug approvals. Ther Innov Regul Sci.

[4] Hunter NL, O’Callaghan KM, Califf RM (2015). Engaging patients across the spectrum of medical product development: view from the US Food and Drug Administration. JAMA.

[5] European Medicines Agency (EMA) Committee for Medicinal Products for Human Use (CHMP) (2020) ICH reflection paper on proposed ICH guideline work to advance patient focused drug development. European Medicines Agency, Amsterdam, The Netherlands. https://www.ema.europa.eu/en/documents/scientific-guideline/ich-reflection-paper-proposed-ich-guideline-work-advance-patient-focused-drug-development_en.pdf. Accessed 31 Jan 2022

[6] Weldring T, Smith SMS (2013). Patient-reported outcomes (PROs) and patient-reported outcome measures (PROMs). Health Serv Insights.

[7] Mercieca-Bebber R, King MT, Calvert MJ, Stockler MR, Friedlander M (2018). The importance of patient-reported outcomes in clinical trials and strategies for future optimization. Patient Relat Outcome Meas.

[8] European Medicines Agency (EMA) Committee for Medicinal Products for Human Use (CHMP) (2016) Appendix 2 to the guideline on the evaluation of anticancer medicinal products in man: the use of patient-reported outcome (PRO) measures in oncology studies. European Medicines Agency, London, United Kingdom. https://www.ema.europa.eu/en/documents/other/appendix-2-guideline-evaluation-anticancer-medicinal-products-man_en.pdf. Accessed 31 Jan 2022

[9] US Food and Drug Administration (2009) Guidance for industry—patient-reported outcome measures: use in medical product development to support labeling claims. Food and Drug Administration, Rockville, MD. https://www.fda.gov/downloads/drugs/guidances/ucm193282.pdf. Accessed 24 Apr 2022

[10] Weiss C, Meehan SR, Brown TM (2021). Effects of adjunctive brexpiprazole on calmness and life engagement in major depressive disorder: *post hoc* analysis of patient-reported outcomes from clinical trial exit interviews. J Patient Rep Outcomes.

[11] Bartrés-Faz D, Cattaneo G, Solana J, Tormos JM, Pascual-Leone A (2018). Meaning in life: resilience beyond reserve. Alzheimers Res Ther.

[12] Rosenblat JD, Simon GE, Sachs GS (2019). Treatment effectiveness and tolerability outcomes that are most important to individuals with bipolar and unipolar depression. J Affect Disord.

[13] Ryff CD (1989). Happiness is everything, or is it? Explorations on the meaning of psychological well-being. J Pers Soc Psychol.

[14] Ryff CD, Keyes CLM (1995). The structure of psychological well-being revisited. J Pers Soc Psychol.

[15] Keyes CLM (1998). Social well-being. Soc Psychol Q.

[16] Leamy M, Bird V, Le Boutillier C, Williams J, Slade M (2011). Conceptual framework for personal recovery in mental health: systematic review and narrative synthesis. Br J Psychiatry.

[17] Morton E, Foxworth P, Dardess P (2022). “Supporting Wellness”: a depression and bipolar support alliance mixed-methods investigation of lived experience perspectives and priorities for mood disorder treatment. J Affect Disord.

[18] Diener E, Emmons RA, Larsen RJ, Griffin S (1985). The Satisfaction With Life Scale. J Pers Assess.

[19] Diener E, Suh EM, Lucas RE, Smith HL (1999). Subjective well-being: three decades of progress. Psychol Bull.

[20] Correll CU, Ismail Z, McIntyre RS, Rafeyan R, Thase ME (2022). Patient functioning and life engagement: unmet needs in major depressive disorder and schizophrenia. J Clin Psychiatry.

[21] Tennant R, Hiller L, Fishwick R (2007). The Warwick–Edinburgh Mental Well-Being Scale (WEMWBS): development and UK validation. Health Qual Life Outcomes.

[22] McIntyre RS, Ismail Z, Watling CP (2022). Patient-reported outcome measures for life engagement in mental health: a systematic review. J Patient Rep Outcomes.

[23] Endicott J, Nee J, Harrison W, Blumenthal R (1993). Quality of Life Enjoyment and Satisfaction Questionnaire: a new measure. Psychopharmacol Bull.

[24] Rush AJ, Gullion CM, Basco MR, Jarrett RB, Trivedi MH (1996). The Inventory of Depressive Symptomatology (IDS): psychometric properties. Psychol Med.

[25] Thase ME, Pedersen AM, Ismail Z et al (2019) Efficacy of adjunctive brexpiprazole in adults with MDD: improvement of patient engagement based on selected items from the Inventory of Depressive Symptomatology Self-Report (IDS-SR) scale. Poster presented at the 32nd annual Psych Congress, San Diego, CA, 3–6 October 2019. https://www.hmpgloballearningnetwork.com/site/pcn/posters/efficacy-adjunctive-brexpiprazole-adults-mdd-improvement-patient-engagement-based-selected. Accessed 31 Jan 2022

[26] Willis GB (2005). Cognitive interviewing: a tool for improving questionnaire design.

[27] Carney DR, Harrigan JA (2003). It takes one to know one: interpersonal sensitivity is related to accurate assessments of others’ interpersonal sensitivity. Emotion.

[28] Inventory of Depressive Symptomatology (Self-Report) (IDS-SR). https://ebbp.org/resources/IDS-SR%20English.pdf. Accessed 31 Jan 2022

[29] Bannigan K, Watson R (2009). Reliability and validity in a nutshell. J Clin Nurs.

[30] Krouwel M, Jolly K, Greenfield S (2019). Comparing Skype (video calling) and in-person qualitative interview modes in a study of people with irritable bowel syndrome—an exploratory comparative analysis. BMC Med Res Methodol.

