# Supplementary material for: Exploring life engagement from the perspective of patients with major depressive disorder: a study using patient interviews
Source: J Patient Rep Outcomes. 2022 Oct 12;6:111. doi: 10.1186/s41687-022-00517-z (PMC9556148; doi:10.1186/s41687-022-00517-z)
Supplement: Supplementary file 1 — Additional file 1. Appendix 1. Worksheet to capture patient ratings of relevance to life engagement for each item in the modified Inventory of Depressive Symptomatology Self-Report (IDS-SR). Appendix 2. Interview guide. [file 41687_2022_517_MOESM1_ESM.pdf]

Supplemental material

Exploring life engagement from the perspective of patients with major depressive disorder: a study using patient interviews

Appendix 1. Worksheet to capture patient ratings of relevance to life engagement for each item in the modified Inventory of Depressive Symptomatology Self-Report (IDS-SR)

Appendix 2. Interview guide

Appendix 1. Worksheet to capture patient ratings of relevance to life engagement for each item in the modified Inventory of Depressive Symptomatology Self-Report (IDS-SR)

	**IDS-SR Items** (each item refers to how you feel over the past seven days)	**1 Not at all Relevant**	**2 A Little Relevant**	**3 Moderately Relevant**	**4 Very Relevant**
1	**Response of Your Mood to Good or Desired Events (e.g., how your mood responds when good events occur)**	☐	☐	☐	☐
2	**Concentration/Decision Making**	☐	☐	☐	☐
3	**View of Myself**	☐	☐	☐	☐
4	**View of My Future**	☐	☐	☐	☐
5	**General Interest (e.g., how interested you are in other people or activities)**	☐	☐	☐	☐
6	**Energy Level**	☐	☐	☐	☐
7	**Capacity for Pleasure or Enjoyment (excluding sex) (e.g., ability to enjoy pleasurable activities)**	☐	☐	☐	☐
8	**Interest in Sex (i.e., interest, not activity)**	☐	☐	☐	☐
9	**Feeling Slowed Down**	☐	☐	☐	☐
10	**Interpersonal Sensitivity (e.g., feeling rejected, criticized, or hurt by others)**	☐	☐	☐	☐
11	**Feeling Sad**	☐	☐	☐	☐
12	**The Quality of Your Mood (e.g., normal, sad; what your mood is like)**	☐	☐	☐	☐
13	**Leaden Paralysis/Physical Energy (e.g., feeling physically weighted down)**	☐	☐	☐	☐
14	**Falling Asleep**	☐	☐	☐	☐
15	**Sleep During the Night**	☐	☐	☐	☐
16	**Waking Up Too Early**	☐	☐	☐	☐
17	**Sleeping Too Much**	☐	☐	☐	☐
18	**Feeling Irritable**	☐	☐	☐	☐
19	**Feeling Anxious or Tense**	☐	☐	☐	☐
20	**Mood in Relation to the Time of Day**	☐	☐	☐	☐
21	**Decreased Appetite**	☐	☐	☐	☐
22	**Increased Appetite**	☐	☐	☐	☐
23	**Decreased Weight (Within the Last Two Weeks)**	☐	☐	☐	☐
24	**Increased Weight (Within the Last Two Weeks)**	☐	☐	☐	☐
25	**Thoughts of Death or Suicide**	☐	☐	☐	☐
26	**Feeling Restless**	☐	☐	☐	☐
27	**Aches and Pains**	☐	☐	☐	☐
28	**Other Bodily Symptoms (e.g., heart pounding fast, blurred vision, sweating, hot and cold flashes, chest pain, heart turning over in chest, ringing in my ears, or shaking)**	☐	☐	☐	☐
29	**Panic/Phobic symptoms**	☐	☐	☐	☐
30	**Constipation/Diarrhea**	☐	☐	☐	☐

Appendix 2. Interview guide

Patient Engagement in Major Depressive Disorder Interview Guide

Introduction

As noted in the consent form, we are working with a pharmaceutical company that develops and markets treatments for major depressive disorder.

Specifically, we are conducting interviews to gather information about patients' experiences with depression, with a focus on improvements associated with successful treatment. We will also ask you to review and provide feedback on the relevance of items in a questionnaire that addresses symptoms of depression.

We are also being asked to pass on to our clients details of adverse events that are mentioned during the course of the research interviews. Although this research interview and what you say will, of course, be treated in confidence, should you raise during the discussion an adverse event in a specific patient, we will need to report this even if it has already been reported by you directly to the company or the regulatory authorities. In such a situation you will be asked whether or not you are willing to waive your confidentiality specifically in relation to that adverse event. Everything else you say during the course of the interview will continue to remain confidential. Do you agree to proceed on this basis?

As we go along, please feel free to speak openly and share your opinions freely. There are no wrong answers here - you are the expert!

Before we begin, do you have any questions?

(Interviewer to ensure that patient has in front of them, the "Engaged with Life" handout and IDS-SR worksheet)

Warm-Up and Word of the Day

Before we begin today, I wanted to take a moment to acknowledge the global crisis we are all dealing with right now—the COVID-19 pandemic.

- How has COVID-19 affected your day-to-day life the most?
- How, if at all, do you think dealing with COVID-19 has affected your mood?

Thank you for taking the time to talk with me about what you are experiencing personally due to COVID-19. I'd like to shift the discussion to your experience with depression.

- At what age were you first diagnosed with depression?
- When was the last time you experienced symptoms of depression for most of the day, nearly every day for at least two weeks? [Refer to screener; note when the last time participant reports experiencing symptoms]
- Tell me what it is like for you, to live with depression?
- How do you feel today? [Probe to understand current mood/anhedonia; if severely depressed mood or flat affect is reported/noted; ask if they think it is affected by COVID-19 and ask if they are OK to continue with the interview; inform the patient that they can take a break or discontinue the interview at any time]
- Has there been a time when you felt better? How do you feel on a good day?

Now, let's talk about words you would use to describe how you feel on good days, bad days, and average days (not good or bad days). Keep in the mind that the words you use to describe the day can relate to how you feel emotionally, physically, your activities, or anything that best describes that day for you.

- On a good day, what words would you use to describe that day? (if needed, ask for 2-3 single, descriptive words)
- What words would you use to describe a bad day? (if needed, ask for 2-3 single, descriptive words)
- What about an average day? Not necessarily good or bad? What words would you use to describe that kind of day? (if needed, ask for 2-3 single, descriptive words)

Patient Engagement Definition and Concept Elicitation

Let's talk more about the good days.

- Have you ever felt like you were "Engaged with Life?" What does that suggest to you?
- **[Look at handout]** Look at the 1-page handout you received. In looking at the quotations in that circle, have you ever felt like that? Tell me about it.

[Probe on the following concepts, if not spontaneously reported]

- How do you **feel** when you're engaged with life?
    - Motivation or energy?
    - Your interest in things? (people, activities intimacy)

- How you feel about yourself?
- Your future?
- View of yourself? (hopefulness/outlook on life, emotional response to positive events, feeling less sensitive or conscientious)

- How, if at all, are your **daily activities** different when engaged with life?
  - More activities (probe on why…energy, being around others, feeling more social, feeling more positive; better/faster decision-making)
  - Different types of activities? How are they different? (probe on why…energy, being around others, feeling more social, feeling more positive, feeling less "brain fog"/ability to focus)
- What about your **involvement or communication with others** when you're engaged with life?
  - Talking more (probe on why…energy, being around others, feeling more social, feeling more positive, feeling less "brain fog"/ability to focus; feeling more interested)
  - Being around people/more people (less alone or isolated; taking things/others less personally [less conscientious])
- What about the way you **think**?
  - Your focus? (probe on why…energy, feeling less "brain fog"; feeling more interested)
  - Thinking more clearly? (probe on why…energy, feeling less "brain fog"; feeling more interested)
  - Your perspective? (future outlook; taking things/others less personally [less conscientious])
  - Your decision-making?
- Does anything else come to mind about what it's like when you feel more engaged with life (that we haven't already talked about)?

IDS-SR Rating Exercise

Now, you are going to look at the document that says "Modified Inventory of Depressive Symptomatology Worksheet" at the top of the page.
[Interviewer to ensure patient is viewing the worksheet]

This worksheet includes 30 items. The first 10 items (2 pages) have been identified as possibly relevant to the concept of "Being Engaged with Life" that we've been talking about.

Please review the first 10 items on the first 2 pages.

- Do you think that they are all related to engagement?
  - Which, if any, of these 10 items do you think are not relevant to "being engaged with life?" (probe on items and reasons why)
- Can you think of any concepts related to engagement that are missing from these 10?

Now, using the 4-point scale at the top of each page, rate each of all 30 items (all pages) for their relevance to "Being Engaged with Life."

(once all 30 items are rated)

Now, tell me about the items you rated as most relevant

Now, let's walk through all the items so you can tell me what you rated for each of them. Perhaps it easiest to tell me all the items you rated as a 4....as a 3..., 2, 1.

(Interviewer to have patient walk through all ratings for recording information)

- Overall, were these items relevant for you? (why/why not)
- Can you think of any concepts that were not on this listing of 30 items that should be included as it relates to being engaged with life? What else?

Wrap-up

Thank you so much for sharing your thoughts and experiences with us today!
